# Impact of general medicine rotation training on the in-training examination scores of 11, 244 Japanese resident physicians: a Nationwide multi-center cross-sectional study

**DOI:** 10.1186/s12909-020-02334-8

**Published:** 2020-11-13

**Authors:** Yuji Nishizaki, Taro Shimizu, Tomohiro Shinozaki, Tomoya Okubo, Yu Yamamoto, Ryota Konishi, Yasuharu Tokuda

**Affiliations:** 1 Medical Technology Innovation Center, Juntendo University, 2-1-1 Hongo, Bunkyo-ku, Tokyo, 113-8421 Japan; 2 Department of Diagnostic and Generalist Medicine, Dokkyo Medical University Hospital, 880 Kitakobayashi, Shimotuga-gun, Mibumachi, Tochigi 321-0293 Japan; 3 Department of Information and Computer Technology, Faculty of Engineering, Tokyo University of Science, 6-3-1 Niijuku, Katsushika-ku, Tokyo, 125-8585 Japan; 4 Research Division, National Center for University Entrance Examinations, 2-19-23 Komaba, Meguro-ku, Tokyo, 153-8501 Japan; 5 Division of General Medicine, Center for Community Medicine, Jichi Medical University School of Medicine, 3311-1 Yakushiji, Shimotsuke, Tochigi 329-0498 Japan; 6 Education Adviser Japan Organization of Occupational Health and Safety, 1-1 KidukiSumiyoshi-cho, Nakahara-ku, Kawasaki-shi, Kanagawa 211-0021 Japan; 7 General Internal Medicine, Muribushi Okinawa for Teaching Hospitals, 3-42-8 Iso, Urasoe-shi, Okinawa 901-2132 Japan

**Keywords:** Basic clinical competency, General medicine, General medicine in-training examination (GM-ITE), Medical education, Resident physician

## Abstract

**Background:**

Although general medicine (GM) faculty in Japanese medical schools have an important role in educating medical students, the importance of residents’ rotation training in GM in postgraduate education has not been sufficiently recognized in Japan. To evaluate the relationship between the rotation of resident physicians in the GM department and their In-Training Examination score.

**Methods:**

This study is a nationwide multi-center cross-sectional study in Japan. Participants of this study are Japanese junior resident physicians [postgraduate year (PGY)-1 and PGY-2] who took the General Medicine In-Training Examination (GM-ITE) in fiscal years 2016 to 2018 at least once (*n* = 11,244). The numbers of participating hospitals in the GM-ITE were 381, 459, and 503 in 2016, 2017, and 2018.The GM-ITE score consisted of four categories (medical interview/professionalism, symptomatology/clinical reasoning, physical examination/procedure, and disease knowledge). We evaluated relationship between educational environment (including hospital information) and the GM-ITE score.

**Results:**

A total of 4464 (39.7%) residents experienced GM department rotation training. Residents who rotated had higher total scores than residents who did not rotate (38.1 ± 12.1, 36.8 ± 11.7, and 36.5 ± 11.5 for residents who experienced GM rotation training, those who did not experience this training in hospitals with a GM department, and those who did not experience GM rotation training in hospitals without a GM department, *p* = 0.0038). The association between GM rotation and competency remained after multivariable adjustment in the multilevel model: the score difference between GM rotation training residents and non-GM rotation residents in hospitals without a GM department was estimated as 1.18 (standard error, 0.30, *p* = 0.0001), which was approximately half of the standard deviation of random effects due to hospital variation (estimated as 2.00).

**Conclusions:**

GM rotation training improved the GM-ITE score of residents and should be considered mandatory for junior residents in Japan.

**Supplementary Information:**

The online version contains supplementary material available at 10.1186/s12909-020-02334-8.

## Background

General medicine (GM) has an important role in health care systems of Western countries. In 1956, the University of Edinburgh opened a Department of GM for the first time in the world. GM physicians have been recognized as specialists of primary care in many European countries with universal health care coverage. The United States health care system has developed a comprehensive GM system, including family medicine for primary care, general internal medicine (GIM) for hospital outpatient care, and hospital medicine for inpatient hospital care [1, 2].

In Japan, a GM department was introduced at Tenri Yorozu Soudan Sho Hospital, a community teaching hospital, in 1976 for the first time [3]. Thereafter, GM departments have been increasing steadily among other community teaching and university hospitals. Physicians in GM departments in Japanese hospitals provide outpatient and/or inpatient care for patients with complex problems and multiple co-morbidities, and who cannot be cared for by sub-specialist physicians.

Although GM faculty members in many Japanese medical schools have important roles as clinician educators for medical students, the importance of GM in a postgraduate residency education has not been sufficiently recognized in Japan [4]. Thus, a 2-year training program for postgraduate junior resident physicians has become mandatory in Japan since 2004, but many junior residents are not required to have rotation training in GM department and are required to have rotations in internal medicine (mostly subspecialty internal medicine divisions), emergency medicine, community medicine, surgery, psychiatry, pediatrics, and obstetrics/gynecology.

Learning objectives in the training program for postgraduate junior resident physicians include many areas related to GM care settings. We believe that GM rotation training has a great effect on the educational achievements of resident physicians. We previously reported a positive association between the presence of a GM department at each teaching hospital and mean scores on the General Medicine In-Training Examination (GM-ITE) among junior residents [5]. However that study was based on an ecological design and did not evaluate the association between GM rotation training and the test scores for individual residents. In the current study, we evaluated the relationship between GM rotation training for residents and their GM-ITE score. We also evaluated the relationship between the GM-ITE scores and factors associated with the educational environment, including time spent on Emergency Department (ED) duties per month, average number of inpatients covered per day, availability and use of online medical resources, and the amount of study time per day.

We have divided the students participating in a GM rotation into three groups, including (1) those with no previous GM experience (i.e., GM rotation) who were trained in hospitals without a GM department (2) those with no previous GM experience who were trained in hospitals that included a GM department, and (3) those with previous GM experience. Teaching hospitals in Japan, regardless of whether or not they have a GM department, all have individual subspecialty divisions of Internal Medicine, including Cardiology, Hematology, Nephrology, and Respiratory Medicine.

## Methods

### Study design

We conducted a nationwide, multi-center, cross-sectional study in Japan. We evaluated the association between the educational environment and the GM-ITE score from the fiscal years 2016 to 2018 among resident physicians in Japanese teaching hospitals. This study was approved by the institutional review board of the Mito Kyodo General Hospital, Mito City, Ibaraki, Japan.

### GM-ITE

The Japan Organization of Advancing Medical Education Program (JAMEP), a non-profit organization, has been implementing the GM-ITE since 2011 as an objective evaluation of the basic clinical competency of junior resident physicians (postgraduate years PGY-1 and PGY-2) [5–9]. The GM-ITE is a multiple-choice knowledge test. In 2016, it consisted of 100 questions. Following revisions in 2017 and 2018, the test has 60 questions.

In line with the early residency objectives of the Japanese Ministry of Health, Labour and Welfare, the GM-ITE covers four areas of basic clinical knowledge: medical interview and professionalism, symptomatology and clinical reasoning, physical examination and procedure, and disease knowledge. The test items relate to the fields of internal medicine, surgery, emergency medicine, pediatrics, obstetrics and gynecology, and psychiatry. Overall, the GM-ITE comprehensively covers all the relevant disciplines with a focus on primary care.

The test content and construct validity have been proven. Upon completing the test, test-takers receive feedback based on the relative scoring of the test participants and a detailed explanation about each question. A sample English question from the GM-ITE is shown in the Additional file 1: Appendix.

### Data collection

We collected information about the educational environment from a self-reporting questionnaire sheet, which included age, future desired specialty after residency, GM rotation, period of internal medicine rotation, ED duties per month, night duties per month, average number of inpatients in charge, use of medical online resources [including UpToDate (www.uptodate.com) [10]], medical journals, medical manuals, medical information websites, study time per day, number of participating case conferences per week, number of participating outside case conferences or lectures, and contribution by the GM department. Hospital information was obtained from the Residency Electronic Information System website [11] and the Foundation for the Promotion of Medical Training website [12]. Regarding the categories of urban cities and local cities in the hospital characteristics, 20 cities designated by the Ministry of Internal Affairs and Communications and in the 23 wards in Tokyo were defined as urban cities and the rest as provincial cities.

### Statistical analyses

Resident physicians were classified into three groups according to their hospitals with or without a GM department and the experience of GM rotation from a self-reporting questionnaire sheet (hospitals without a GM department did not offer GM rotation). We compared the GM-ITE total scores between the GM department/rotation groups by using linear multilevel regression models adjusted for hospital-level information (hospital location and hospital type) and resident-level information (sex, deviation value of graduating school in 2018, ED duties per month, average number of inpatients in charge, UpToDate use, study time per day, and year of GM-ITE) as well as hospitals as random normal intercepts. Residents with missing data for any variable were excluded from the multivariate analysis. All analyses were performed by using SAS version 9.4 software (SAS Institute Inc., Cary, NC). We interpreted *p*-values as indicative of compatibility between the data and the “no-difference between that variable’s levels” hypothesis under the assumed statistical model.

## Results

Throughout Japan, 381, 459, and 503 hospitals participated in the GM-ITE in 2016, 2017, and 2018, and the numbers of GM-ITE participants were 4568, 5593, and 6133, respectively, for each year. If consent for utilizing the GM-ITE score could not be obtained, those data were excluded from the analyses. If a resident physician took the examination twice (in PGY-1 and PGY-2), we excluded the data from PGY-1. Finally, the data of 11,244 resident physicians were used for analyses.

Summarized hospital information, resident information, and GM-ITE scores are shown in Table 1. A total of 11,244 residents (67.7% men) were retrospectively analyzed. Among the hospitals, 26.8% were urban and 88.8% were community hospitals. The average total GM-ITE scores were 37.3 ± 11.9 and 5.9 ± 5.4 for medical interview and professionalism, 10.6 ± 2.5 for symptomatology and clinical reasoning, 10.3 ± 2.5 for physical examination and procedure, and 10.5 ± 4.2 for disease knowledge.

**Table 1:** Educational environment, characteristics of the residents, and GM-ITE scores

Variables	All	GM department yes		GM department no	*p-*value^*1^
		GM rotation yes	GM rotation no	GM rotation no	
**Hospital information**					
Location					
Urban area, n (%)	3013 (26.8)	1197 (26.8)	1283 (26.1)	533 (28.7)	0.9256
Rural area, n (%)	8231 (73.2)	3267 (73.2)	3641 (73.9)	1323 (71.3)	
Hospital type					
Community hospital (incl. University branch hospital), n (%)	9990 (88.8)	4009 (89.8)	4150 (84.3)	1831 (98.7)	0.0056
University hospital, n (%)	1254 (11.2)	455 (10.2)	774 (15.7)	25 (1.3)	
**Resident information**					
Male, n (%)	7611 (67.7)	3075 (68.9)	3294 (66.9)	1242 (66.9)	0.1981
Female, n (%)	3633 (32.3)	1389 (31.1)	1630 (33.1)	614 (33.1)	
Deviation value, mean ± SD	66.4 ± 2.2	66.3 ± 2.2	66.4 ± 2.2	66.6 ± 2.2	0.4448
ED duties per month					
0 per month, n (%)	347 (3.1)	127 (2.8)	180 (3.7)	40 (2.2)	0.0163 ^*2^
1 to 2 per month, n (%)	1543 (13.7)	510 (11.4)	878 (17.9)	155 (8.4)	
3 to 5 per month, n (%)	7779 (69.3)	3098 (69.5)	3235 (65.8)	1446 (78)	
More than 6 per month, n (%)	1473 (13.1)	693 (15.5)	575 (11.7)	205 (11.1)	
Unknown	84 (0.7)	30 (0.7)	47 (1)	7 (0.4)	
Average number of inpatients in charge					
0 to 4, n (%)	1776 (15.9)	682 (15.3)	784 (16)	310 (16.8)	0.708 ^*2^
5 to 9, n (%)	6650 (59.4)	2622 (59)	2947 (60.1)	1081 (58.4)	
10 to 14, n (%)	1812 (16.2)	725 (16.3)	770 (15.7)	317 (17.1)	
More than 15, n (%)	615 (5.5)	310 (7)	245 (5)	60 (3.2)	
Unknown, n (%)	345 (3.1)	108 (2.4)	155 (3.2)	82 (4.4)	
UpToDate use					
No, n (%)	5683 (72.4)	1948 (64.4)	2688 (76.7)	1047 (79.6)	<.0001
Yes, n (%)	2162 (27.6)	1077 (35.6)	816 (23.3)	269 (20.4)	
Study time					
None, n (%)	655 (5.9)	249 (5.6)	308 (6.3)	98 (5.3)	0.0536
0 to 30 min, n (%)	4022 (36)	1567 (35.2)	1864 (38.1)	591 (32)	
31 to 60 min, n (%)	4457 (39.8)	1762 (39.6)	1875 (38.3)	820 (44.4)	
61 to 90 min, n (%)	1600 (14.3)	672 (15.1)	663 (13.6)	265 (14.3)	
More than 91 min, n (%)	451 (4)	198 (4.5)	180 (3.7)	73 (4)	
Year					
2016, n (%)	2748 (24.4)	1089 (24.4)	1219 (24.8)	440 (23.7)	0.1182
2017, n (%)	3251 (28.9)	1380 (30.9)	1395 (28.3)	476 (25.6)	
2018, n (%)	5245 (46.6)	1995 (44.7)	2310 (46.9)	940 (50.6)	
GM-ITE score					
Total, mean ± SD	37.3 ± 11.9	38.1 ± 12.1	36.8 ± 11.7	36.5 ± 11.5	0.0038
Medical interview and professionalism, mean ± SD	5.9 ± 5.4	6.1 ± 5.6	5.8 ± 5.3	5.7 ± 5.2	0.204
Symptomatology and clinical reasoning, mean ± SD	10.6 ± 2.5	10.8 ± 2.6	10.4 ± 2.5	10.4 ± 2.4	0.0002
Physical examination and procedure, mean ± SD	10.3 ± 2.5	10.5 ± 2.5	10.1 ± 2.5	10.1 ± 2.5	0.0006
Subspecialties, mean ± SD	10.5 ± 4.2	10.8 ± 4.2	10.4 ± 4.2	10.2 ± 4.1	0.0069

*GM* general medicine, *GM-ITE* general medicine in-training examination, *SD* standard deviation, *ED* emergency department

^*1^ = *p*-values from generalized estimating equations of univariable logistic (binary data), proportional odds (multicategory data), or linear (continuous data) models with clustering hospitals. ^*2^ = Excluding data in “Unknown” category

A total of 4464 (39.7%) residents experienced rotation training in the GM department. Residents who rotated in the GM department had higher total scores than residents who did not rotate. The results were 38.1 ± 12.1, 36.8 ± 11.7, and 36.5 ± 11.5 for residents who experienced GM rotation training, those who did not experience GM rotation in hospitals with a GM department, and those who did not experience GM rotation in hospitals without a GM department (*p* = 0.0038).

The association between GM department/rotation remained after multivariable adjustment in the multilevel model (Table 2): the score difference between residents who experienced GM rotation training and those who did not in hospitals without a GM department was estimated as 1.18, with a standard error of 0.30 (*p* = 0.0001), which was about half of the standard deviation of random effects due to hospital variation (estimated as 2.00). The analysis also showed that community hospital, male sex, deviation value, ED duties per month (three to five per month vs. zero per month: estimated coefficient, 1.22; standard error, 0.40; *p* = 0.0022), average number of inpatients in charge (> 15 vs. zero to four: estimated coefficient, 1.18; standard error, 0.40; *p* = 0.0036), UpToDate use (estimated coefficient, 1.48; standard error, 0.16; *p* < 0.0001), study time (61–90 min vs. none: estimated coefficient, 1.65; standard error, 0.39; *p <* 0.0001), test year, and GM rotation training were associated with the total GM-ITE score.

**Table 2:** Factors related to GM-ITE score (multilevel analysis)

Variables	Estimated Coefficient	Standard Error	*p*-value
**Hospital information**			
Rural area (vs. Urban area)	0.3027	0.2795	0.279
University hospital [vs. Community hospital (incl. University branch hospital)]	−1.7672	0.5052	0.0005
**Resident information**			
Sex			
Male	0		
Female	−0.3344	0.1511	0.0269
Deviation value	0.2904	0.03638	< 0.0001
ED duties per month			
0 per month	0		
1 to 2 per month	0.6283	0.4287	0.1428
3 to 5 per month	1.2235	0.4003	0.0022
More than 6 per month	1.1435	0.4499	0.0111
Unknown	0.1298	0.8869	0.8836
Average number of inpatients in charge			
0 to 4	0		
5 to 9	1.0089	0.2026	< 0.0001
10 to 14	0.9146	0.2646	0.0006
More than 15	1.1861	0.4068	0.0036
Unknown	0.3277	0.4227	0.4382
UpToDate use (vs. Not used)	1.4842	0.1663	< 0.0001
Study time per day			
None	0		
0 to 30 min	0.5612	0.2873	0.0508
31 to 60 min	1.1379	0.29	< 0.0001
61 to 90 min	1.7196	0.3308	< 0.0001
More than 91 min	2.2147	0.4613	< 0.0001
Year			
2016	0		
2017	−22.981	0.1846	< 0.0001
2018	−24.2165	0.1651	< 0.0001
**Combination of resident and hospital information**			
GM department and rotation			
Did not experience GM rotation in hospitals without GM department	0		
Did not experience GM rotation in hospitals with GM department	0.9423	0.3065	0.0021
Experienced GM rotation	1.1848	0.3045	0.0001

*GM* general medicine, *GM-ITE* general medicine in-training examination, *ED* emergency department

Table 3 includes the results of subgroup multilevel regression analyses that evaluate the relationship between the GM rotation and the GM-ITE score (Table 3). Overall, we found no obvious difference in relationships between the GM rotation and the GM-ITE score with respect to each subgroup except for hospital type (community hospitals vs. university hospitals).

**Table 3:** Results of subgroup analysis

Subgroup	GM rotation/GM department	N	Estimated Coefficient	SE	*P*-value
Urban area	no/no	533	0		
	no/yes	1283	0.9997	0.656	0.1277
	yes/yes	1197	1.3449	0.6458	0.0374
Rural area	no/no	1323	0		
	no/yes	3641	0.9057	0.3448	0.0086
	yes/yes	3267	1.1051	0.3435	0.0013
Community hospital	no/no	1831	0		
	no/yes	4150	0.9369	0.3121	0.0027
	yes/yes	4009	1.0941	0.3098	0.0004
University Hospital	no/no	25	0		
	no/yes	774	2.9894	2.2138	0.1773
	yes/yes	455	3.6334	2.1959	0.0984
Male	no/no	1242	0		
	no/yes	3294	0.7854	0.3533	0.0263
	yes/yes	3075	1.1612	0.3513	0.001
Female	no/no	614	0		
	no/yes	1630	0.9223	0.4261	0.0305
	yes/yes	1389	1.206	0.4308	0.0052
Deviation value <= 65.0	no/no	892	0		
	no/yes	2570	0.7941	0.3787	0.036
	yes/yes	2413	1.0696	0.3765	0.0045
Deviation value > = 65.1	no/no	964	0		
	no/yes	2354	0.9123	0.4119	0.0269
	yes/yes	2051	1.2676	0.4137	0.0022
ED duty: 0 per month	no/no	40	0		
	no/yes	180	0.9343	1.3604	0.4935
	yes/yes	127	−0.04607	1.3895	0.9736
ED duty: 1 to 2 per month	no/no	155	0		
	no/yes	878	1.2616	0.8098	0.1196
	yes/yes	510	1.6789	0.8348	0.0446
ED duty: 3 to 5 per month	no/no	1446	0		
	no/yes	3235	0.8094	0.3388	0.0169
	yes/yes	3098	1.097	0.3371	0.0011
ED duty: more than 6 per month	no/no	205	0		
	no/yes	575	0.629	0.7102	0.376
	yes/yes	693	1.4192	0.7069	0.045
Average number of inpatients in charge: 0 to 4	no/no	310	0		
	no/yes	784	1.0523	0.5967	0.0781
	yes/yes	682	0.8281	0.6093	0.1744
Average number of inpatients in charge: 5 to 9	no/no	1081	0		
	no/yes	2947	0.9504	0.3662	0.0095
	yes/yes	2622	1.3387	0.3648	0.0002
Average number of inpatients in charge: 10 to 14	no/no	317	0		
	no/yes	770	0.01113	0.6356	0.986
	yes/yes	725	0.8508	0.6402	0.1842
Average number of inpatients in charge: more than 15	no/no	60	0		
	no/yes	245	−0.02175	1.1874	0.9854
	yes/yes	310	0.1001	1.1793	0.9324
UpToDate non-user	no/no	1047	0		
	no/yes	2688	0.7996	0.312	0.0104
	yes/yes	1948	0.8833	0.3143	0.005
UpToDate user	no/no	269	0		
	no/yes	816	1.048	0.576	0.069
	yes/yes	1077	1.9872	0.5635	0.0004
Study time: none	no/no	98	0		
	no/yes	308	0.9609	0.9799	0.3277
	yes/yes	249	−0.1344	1.0034	0.8936
Study time: 0 to 30 min	no/no	591	0		
	no/yes	1864	1.1565	0.4112	0.0049
	yes/yes	1567	1.5432	0.4161	0.0002
Study time: 31 to 60 min	no/no	820	0		
	no/yes	1875	0.6662	0.4023	0.0979
	yes/yes	1762	1.4065	0.4027	0.0005
Study time: 61 to 90 min	no/no	265	0		
	no/yes	663	0.2159	0.6385	0.7354
	yes/yes	672	0.8812	0.6487	0.1748
Study time: more than 91 min	no/no	73	0		
	no/yes	180	0.3576	1.3614	0.7935
	yes/yes	198	0.9663	1.3499	0.4761

*ED* emergency department

Each multilevel regression analysis adjusted for variables indicated in Table 2

## Discussion

We found a positive relationship between the experience of GM rotation training and the total GM-ITE score. The contribution for improving the GM-ITE score ranged from highest to lowest as follows: experienced GM rotation training > did not experience GM rotation training in hospitals with a GM department > did not experience GM rotation training in hospitals without a GM department. These results suggest that educational achievement in junior residents could be improved by their GM rotation training. We also found the several significant factors associated with the educational environment that had an impact on the GM-ITE score, including ED duties (shifts per month), average number of inpatients covered per day, availability and use of online medical resources, and amount of study time per day. Although amount of study time and use of effective learning materials are important factors with direct impact on test preparedness, we found no specific change in relationships between the GM rotation and the GM-ITE score across subgroups of these factors.

We have previously reported that residents in teaching hospitals with higher mean GM-ITE scores were associated with the presence of a GIM or GM department [5]. However, that study was an investigation with a small sample size of 206 residents at 21 hospitals. Furthermore, because there was no questionnaire survey on the educational environment, the association between GM rotation and GM-ITE score was not verified. In this study, we used a larger sample size and more directly evaluated the effect of GM rotation training on educational achievements/competency by specifically investigating the relationship between GM rotation training and the GM-ITE score for residents.

We believe bedside teaching by general physicians provides greater effectiveness of clinical education. The GM department covers all areas of medicine with a broad view to patient care and does not focus on a single organ system, so training programs involving GM rotation positively affect the GM-ITE score. Moreover, general physicians are good at teaching essential elements for basic clinical competency, such as clinical ethics, communication, physical examination, clinical reasoning, and professionalism [13–15].

Compared with Western countries, Japan has a shorter history of developing GM departments in hospitals, so these have not been recognized as a required department for educational rotation of junior residents [16–18]. However, since there is a broad overlap of core competencies between GM physicians and junior residents, rotation training in this department is highly desirable. From 2018, the designation of “general physician” has been newly acknowledged as a specialist in a basic area of the Japanese medical specialty system [19, 20]. It is hoped that this change will serve as an opportunity to introduce GM departments in all teaching hospitals throughout Japan.

The hospitalist plays a crucial role in improving the quality of resident physician education [21]. As in the United States, Japanese hospitals should have a GM department at the center of the medical ward, and it is necessary to build a system in which general physicians take care of the patient along with the resident in the medical ward and have the option to immediately consult with sub-specialist physicians if necessary. Further, sub-specialist physicians should be able to consult general physicians in order to facilitate a smooth diagnosis [2, 22].

We found that resident physicians with ED duty three to five times per month were associated with the highest GM-ITE scores relative to those with no ED duty per month. Residents with three to five instances of ED duty per month had higher GM-ITE scores than residents with more than six instances per month. The results revealed a trend similar to that of a previous study we reported in 2015 [6]. An appropriate workload in ED duty may develop better competencies and lead to better educational achievements among resident physicians. On the other hand, an excessive workload may cause “burn out” or more medical errors [23–25]. Burnout is a psychological syndrome that is experienced frequently by medical residents. It consists of emotional exhaustion, depersonalization and reduced feelings of personal accomplishment [26]. Trigger factors for burnout among emergency physicians and emergency medicine residents include non-patient-related problems (such as large administrative burdens) in addition to personal and interpersonal issues [27]. Shanafelt et al. reported differences in burnout by specialty; the highest rates of burnout are reported among physicians on the front lines of medical care (i.e., family medicine, general internal medicine, and emergency medicine). This group also reported that, compared with a probability-based cohort sample of 3442 working U. S. adults, physicians were more likely to have symptoms of burnout (37.9% vs 27.8%) and to be dissatisfied with their current level of work-life balance (40.2% vs 23.2%) [28]. Therefore, we should require continual awareness of the optimized workload balance in ED duties to provide a safer educational environment for resident physicians.

We showed that resident physicians in charge of > 15 inpatients had higher GM-ITE scores than residents in charge of 0 to 4 inpatients. We also demonstrated a relationship between appropriate study time and higher GM-ITE score. The explanation for this observation lies in the following witty remark by William Osler: “To study the phenomena of disease without books is to sail an uncharted sea, while to study books without patients is not to go to sea at all” [29, 30].

UpToDate use was associated with higher GM-ITE scores. The results coincided with those of our previous report [6]. Physicians need to effectively collect clinical evidence for a short period in a clinical situation. Self-directed reading of an electronic knowledge resource have been found previously to have statistically and educationally significant independent associations with medical knowledge acquisition [31]. In addition, efficient collection of clinical evidence by using UpToDate could improve not only medical knowledge but also prognoses of patients [32, 33]. We expect resident physicians to appropriately use evidence-based electronic resources for bedside and clinical decision making.

Brint and Cantwell reported that more time spent focused on learning results in improved performance on examinations [34]; reports from other groups revealed that use of online resources resulted in improved student performance [35, 36]. Our findings are consistent with the results of these earlier studies.

We found that the group of “no experience of GM rotation in hospitals with GM department” tended to obtain the higher GM-ITE score than the group of “no experience of GM rotation in hospitals without GM department”. Even if junior resident physicians had no previous GM experience, GM departments tend to have an overall profound educational impact on resident physicians. GM departments typically include faculty development programs that provide outreach to all departments. GM physicians are also among the most likely to provide resident physicians lectures and conferences that cover topics that are critical for clinical training including ethics, communication skills, professionalism, and evidence-based medicine.

As shown in Table 3, there was no apparent difference in relationship between having the experience of a GM rotation and the GM-ITE score with respect to location (i.e., urban area vs. rural area). On the other hand, there were significant differences in the relationship between hospital type subgroups (community hospitals vs. university hospitals), with the university hospitals having a greater GM rotation and the GM-ITE score associations than community hospitals. The estimated coefficient for “having experienced a GM rotation” was 1.1 in community hospitals and 3.6 in university hospitals. We hypothesize that this result may reflect differences in the environment related to primary care education and thus a higher educational impact of the GM rotation.

The present study revealed a positive association between having participated in a GM rotation and GM-ITE score. In addition to the positive impact of a GM rotation, we think that the potential negative impact of limited training in specialized medical departments should not be overlooked. In modern clinical practice, physicians are asked to care for patients with complex medical problems and multiple co-morbidities. As such, physicians who are focused purely on one organ or organ system may have a more narrow vision, and thus may provide a limited educational view for junior resident physicians. Furthermore, learning objectives for postgraduate junior resident physicians include aspects such as ethics, communication skills, professionalism, and evidence-based medical practice, among other more general topics. It is unlikely that a medical sub-specialist will have the knowledge or interest in taking charge of so wide a range of educational requirements. Given this situation, we feel strongly that further enrichment of the education system with a strong focus on GM departments will ensure that the next generation of physicians is fully equipped to deal with a wide range of problems.

However, there are other points that require some future consideration. Sub-specialists may need to put in more effort towards identifying cross-disciplinary solutions for both social and medical problems, including the doctor–patient relationship, problems associated with mental and psychological well-being as well as social welfare, and various approaches that feature input from those knowledgeable in the behavioral sciences. Furthermore, sub-specialists may need to contribute toward patient-centered health education regarding smoking, drinking alcohol, and drug abuse. The sub-specialists will need to foster and nurture sensitivity toward these problems; we recognize that this will be a substantial paradigm-shift from the current disease-oriented approach [3].

We were unable to identify any specific factors underlying the gender differences in the GM-ITE scores. This result did not necessarily mean that female resident physicians possess less knowledge or fewer skills than do their male resident physicians. Additional analysis with representative data will be needed to address this issue.

Our study had several limitations. First, we did not include all initial training participants belonging to teaching hospitals in Japan. The number of PGY-1 plus PGY-2 resident physicians was approximately 18,000, but about one-third of resident physicians participate in the GM-ITE. Therefore, our findings might not be generalizable to all residents throughout Japan.

Second, we did not evaluate the residents’ baseline GM-ITE score. For a more precise measurement of the impact of general medicine rotation training on the GM-ITE score, the baseline GM-ITE score should be adjusted.

Third, we did not take the participants’ medical school experiences into consideration. The learning experiences of GM training depend on one’s medical school education. This may have affected the study results.

The fourth limitation is the season of the GM rotation, that is, the GM-ITE scores are affected if the season of the GM rotation is close to the time of testing. We were not able to adjust this factor.

Finally, the study variables did not include some that might have affected the results. For example, the number of supervising physicians belonging to a GM department and the years of clinical experience of the supervising physicians and contents of their educational programs could affect the learned competencies and GM-ITE scores of the residents. Future studies are needed to confirm the direct relationship between GM rotation training and GM-ITE scores after adjusting for the variables mentioned above.

## Conclusion

GM rotation training led to improvement in the GM-ITE score of residents. Therefore, mandatory GM rotation training should be considered for junior residents in Japan.

## Supplementary Information

**Additional file 1.** Appendix

## Data Availability

The data sets will not be publicly available because informed consent of participants in each hospital do not allow for such publication. The corresponding author will respond to requests on data analyses.

## Abbreviations

ED: Emergency department
GIM: General internal medicine
GM: General medicine
GM-ITE: General Medicine In-Training Examination
JAMEP: Japan Organization of Advancing Medical Education Program
PGY: Postgraduate year
