# Supplementary material for: Impact of general medicine rotation training on the in-training examination scores of 11, 244 Japanese resident physicians: a Nationwide multi-center cross-sectional study
Source: BMC Med Educ. 2020 Nov 13;20:426. doi: 10.1186/s12909-020-02334-8 (PMC7666491; doi:10.1186/s12909-020-02334-8)
Supplement: Supplementary file 1 — Additional file 1. Appendix [file 12909_2020_2334_MOESM1_ESM.docx]

Appendix

**Question**

A 67-year-old man is evaluated for a rash of 2 days’ duration. It is located over the right forehead and has vesicles and pustules, and his right eyelid is swollen. The rash was preceded by tingling in the area and is now associated with aching pain. He denies pain in other sites and change in vision. He has hypertension and takes amlodipine. He denies any history of drug allergy. He used to work as a high school teacher but is retired now. He is a former smoker. He drinks 1-2 glasses of beer almost every night.

On physical examination, vital signs are normal. He appears in no distress. There are clusters of pustules and clear vesicles over his forehead. The upper eyelid is swollen. There is no conjunctival injection. His visual acuity is normal. The remainder of the examination is unremarkable. He is given analgesics.

Which is the most appropriate management?

1. Start valacyclovir and refer him to an ophthalmologist
2. Start a topical steroid ointment
3. Order a polymerase-chain-reaction (PCR) assay for varicella-zoster virus DNA from the base of the lesions
4. Start valacyclovir

**Answer**

(1) Start valacyclovir and refer him to an ophthalmologist

**Commentary**

This 67-year-old man with hypertension has presented with sudden-onset a vesicular and pustular rash on one side of the forehead. The ophthalmological features and distribution of the rash indicate herpes zoster. Herpes zoster occurs following the reactivation of herpes zoster virus dormant in the cerebral nerves or dorsal root ganglion, spreading along the dermatome innervated by the affected nerve. Age-related decline in cellular immunity is a particularly well-known risk factor. Herpes zoster is clinically diagnosable if the symptoms are classical. Herpes zoster ophthalmicus is a concern in this patient because the rash affects the first (ophthalmic) branch of the trigeminal nerve. In herpes zoster ophthalmicus, the normal rash often precedes ocular symptoms by a few days. Caution is required when the rash appears in the area innervated by the nasociliary nerve, which runs from the inside of the medial canthus to the tip of the nose (Hutchinson’s sign). Herpes zoster ophthalmicus should be managed with an analgesic and antiviral drug and referral to an ophthalmologist. Complications that threaten visual acuity can occur if the virus invades the cornea. Local application of a steroid ointment would provide no benefit.

**References**

1. Cohen JI. Herpes zoster. N Engl J Med. 2013; 369: 255-63. PMID: 23863052.
2. Opstelten W, Zaal MJ. Managing ophthalmic herpes zoster in primary care. BMJ. 2005; 331: 147-51. PMID: 16020856.
